# Relapsed and refractory yolk sac tumor of the peritoneum (mesentery): A case report and literature review

**DOI:** 10.3389/fonc.2022.928234

**Published:** 2022-08-09

**Authors:** Xue Zhou, Lanbo Zhao, Xue Feng, Zhenni Pan, Yadi Bin, Siyi Zhang, Min Li, Miao Guo, Huilian Hou, Qiling Li

**Affiliations:** ^1^ Department of Obstetrics and Gynecology, First Affiliated Hospital of Xi’an Jiaotong University, Xi’an, China; ^2^ Department of Obstetrics and Gynecology, Tangdu Hospital of the Fourth Military Medical University, Xi’an, China; ^3^ Department of Pathology, First Affiliated Hospital of Xi’an Jiaotong University, Xi’an, China

**Keywords:** recrudescence, yolk sac tumor, peritoneum, boanmycin, literature review

## Abstract

**Background:**

Extragonadal yolk sac tumor (YST) of peritoneum is a rare malignancy.

**Case Description:**

A 37-year-old Chinese woman was admitted to hospital with a 3-month abdominal pain 4 years ago. Alpha-fetoprotein was 228,499.0 ng/mL. Computed tomography scan revealed a massive mass in the left lower abdomen. Exploratory laparotomy exposed a huge mesenteric mass. Then, mesenteric tumor resection, partial sigmoidectomy, and single-lumen fistula of sigmoid colon were performed. Postoperative pathologic diagnosis reported a stage IV mesenteric YST. After surgery, the patient received 6 courses of BEP (bleomycin, etoposide, and cisplatin) chemotherapy. Seven months later, the patient underwent stoma reversion of sigmoid colon and received another 2 courses of BEP chemotherapy. Three months after the last chemotherapy, liver metastases were diagnosed. She subsequently underwent 3 surgeries, radiotherapy for liver metastases, and multiple tiers of palliative chemotherapies, including TP (docetaxel and carboplatin), VIP (ifosfamide, cisplatin, and etoposide), TIP (paclitaxel, ifosfamide, and cisplatin), and so on. After the third surgery (left hepatic lesion resection and right iliac lymph node resection), she received 4 cyclic chemotherapies of BEP´ (boanmycin, etoposide, and cisplatin) without pulmonary toxic side effects.

**Conclusion:**

Postoperative histopathology and immunohistochemistry are gold standards for the diagnosis of peritoneal YST. The standard first-line treatment is surgery plus BEP chemotherapy. Second-line therapy regimens and above, including VIP and TIP, improve the prognosis of recurrent germ cell tumors. This relapsed and refractory patient with peritoneal YST benefits from the secondary BEP´ chemotherapy.

## Introduction

Yolk sac tumor (YST, endodermal sinus tumor) is the third common highly-malignant germ cell tumor, which often occurs in testes and ovaries (1–3). YST is a combination of cystic and solid mass, and it is mostly unilateral, large, and well-encapsulated (4). YST has a variety of histological appearances, of which reticular or microcystic pattern is the most common (4–7). Serum and immunohistochemical staining are positive for alpha-fetoprotein (AFP) in most YST patients (8, 9). Currently, the overall cure rate for YST patients after surgery and chemotherapy is 80% (3, 10).

However, the tumor also rarely occurs in the extragonadal locations, such as the sacrococcyx, mediastinum, brain, stomach, vagina, retroperitoneum, and liver (7, 11, 12). Peritoneum is the largest serous membrane in the human body, being composed of mesothelial cells and connective tissue with elastic fibers (13). The structure of the peritoneum contains omentum, mesangium, ligaments, peritoneal folds, peritoneal recesses, and depressions (14). The reported primary tumors of peritoneum are scarce, including extraovarian primary peritoneal carcinoma, malignant mesothelioma, multicystic mesothelioma, leiomyosarcomas, leiomyomatosis peritonealis disseminata, and desmoplastic small round cells tumor (15, 16). Peritoneum is an extremely rare location for primary YST (7). Currently, the standard first-line chemotherapy for extraglandular YST is BEP (bleomycin, etoposide, and cisplatin) (1), in which bleomycin has a cumulative lifetime dose and pulmonary toxicity. Boanmycin, a bleomycin analog, has relatively lower pulmonary toxicity (17). To our knowledge, there is no review on YST originating from the peritoneum. We reported a case of relapsed and refractory peritoneal mesenteric YST that benefited from secondary BEP’ (boanmycin, etoposide, and cisplatin) chemotherapy and performed a literature review.

## Case report

**Figure 1:**
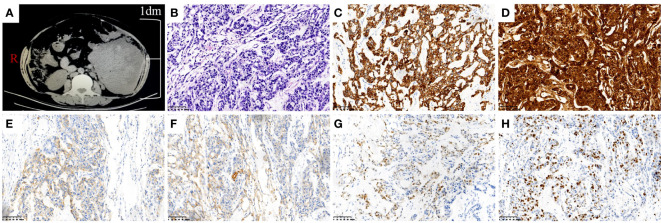
Image picture and pathologic images of mesenteric YST. **(A)** CT scan of mass in the left lower quadrant. **(B)** Hematoxylin-eosin staining of the mesenteric YST (×200). **(C)** Immunohistochemical staining of CK was strong positive(×200). **(D)** Immunohistochemical staining of AFP was strong positive(×200). **(E)** Immunohistochemical staining of CD117 was weakly positive (×200). **(F)** Immunohistochemical staining of PALP was weakly positive (×200). **(G)** Immunohistochemical staining of CDX2 was weakly positive (×200). **(H)** Immunohistochemical staining of Ki-67 was 50% positive (×200).

A 34-year-old woman, para 2-0-0-2, visited the Second Affiliated Hospital of Xi’an Jiaotong University because of a 3-month abdominal pain 4 years ago. She developed intermittent left abdominal pain and discomfort at 6-month of pregnancy but ignored this symptom. On pelvic examination, a hard immovable mass about 150 × 130 mm was palpable on the left lower quadrant of abdomen. The pregnant fundus of the uterus was located four fingers above the pubic symphysis with serum AFP being 228,499.0 ng/mL (normal level: less than 7.0 ng/mL) and serum cancer antigen 125 (CA 125) being 167.4 U/mL (normal level: less than 36.0 U/mL). Computed tomography (CT) scan revealed a soft tissue mass of approximately 143 × 151 × 125 mm in the left lower quadrant with ascites (**Figure 1A**). There were multiple calcifications in the liver and spleen, and no obvious abnormal density in the bilateral uterine appendages with normal chest X-ray and other examinations.

Combining the above clinical manifestations and examination results, her initial diagnosis was abdominal tumor. Explorative laparotomy was performed and a mesenteric mass, about 170 × 150 × 75 mm, was discovered. The mass was located between the sigmoid colon and lateral peritoneum with 1,000 mL ascites. The upper, lower, and outer boundaries of the mass were the lower level of the colon and kidney, the pelvic entrance, and the lateral peritoneum, respectively. The inner side was closely connected with the sigmoid colon and descending colon. Tumor resection, partial sigmoidectomy, and single-lumen fistula of sigmoid colon were performed on 28 June 2018. Her postoperative pathology was YST of mesenteric (**Figure 1B**). The results of immunohistochemistry were as follows: CK (+++), AFP (+++), CD117 (+), PALP (+), CDX2 (+), CD34 (+), EMA (+), CD56 (+), CD20 (+), Ki67 (50% +), and other immune markers were negative (**Figures 1C–G**). On the 15th postoperative day, BEP adjunct chemotherapy consisting of bleomycin (15 mg iv for 3 consecutive days), etoposide (100 mg iv for 4 consecutive days), and cisplatin (30 mg iv for 3 consecutive days) was performed every 3 weeks over six cycles. Serum AFP had fallen to normal after the fourth cycle of chemotherapy. The last chemotherapeutic date was 28 October 2018. Subsequently, she followed up with serum AFP and abdominal CT scan regularly.

She underwent the second operation — stoma reversion of sigmoid colon on 12 May 2019. During the operation, two 10 mm masses were excised with negative serum AFP. The pathology was the same as before. Two cycles of BEP adjunct chemotherapy (dosage: the same as before) were performed again.

**Figure 2:**
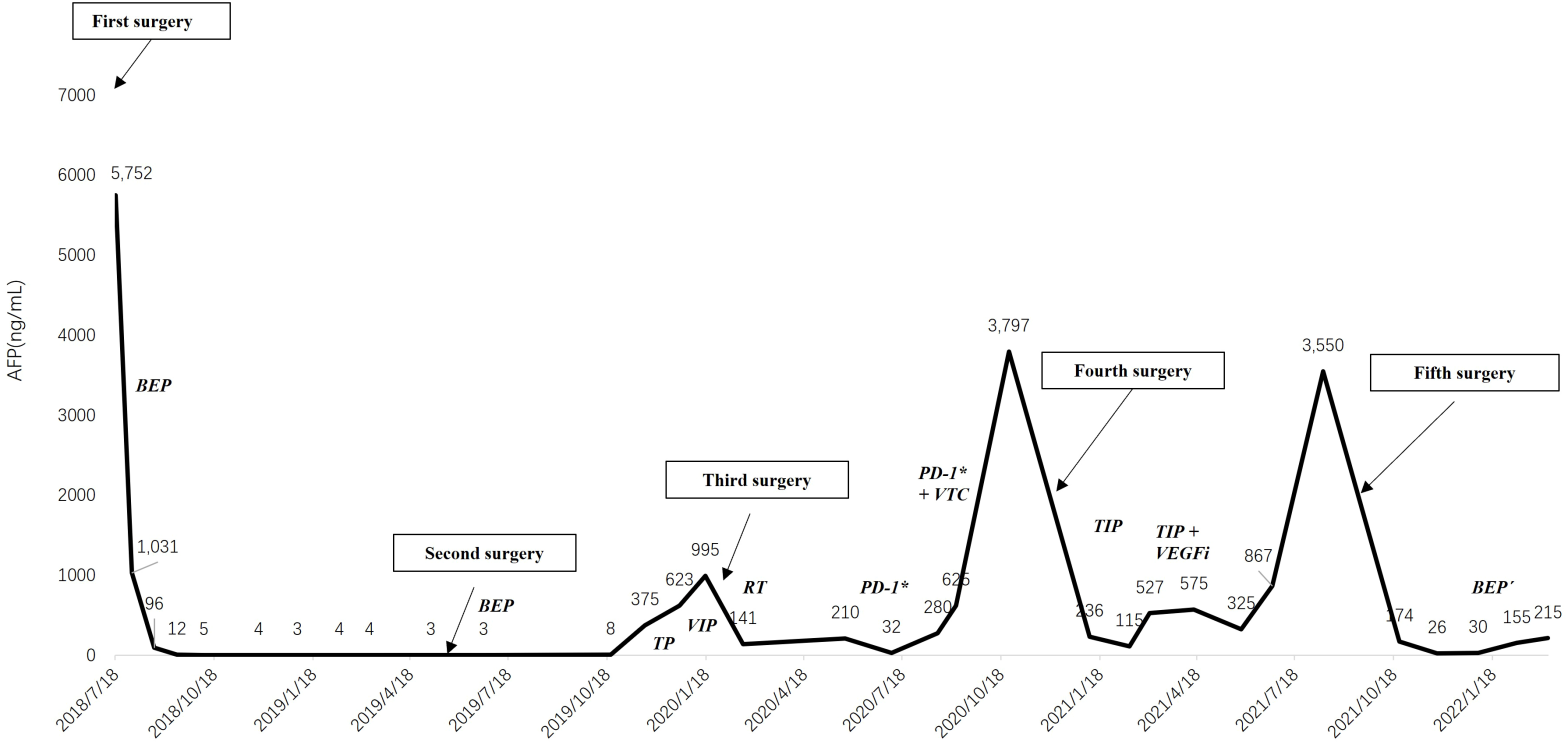
The changing trend of serum alpha-fetoprotein. BEP, bleomycin, etoposide and cisplatin; TP, docetaxel and carboplatin; VIP, ifosfamide, cisplatin and etoposide; RT, radiotherapy; PD-1*, tislelizumab; VTC, vinorelbine tartrate capsules; TIP, paclitaxel, ifosfamide and cisplatin; VEFGi, bevacizumab. *: Tislelizumab is an anti-programmed cell death protein-1 (PD-1) antibody.

On 21 October 2019, the patient received an abdominal CT scan showing multiple low-density masses in the right liver, and then underwent a needle biopsy. The pathology showed metastatic malignant tumor. Because the chemotherapy-free interval was less than 6 months, the chemotherapy regimen was changed to second-line palliative chemotherapy, namely TP (docetaxel 75 mg/m^2^ iv, carboplatin 350 mg/m^2^ iv). As serum AFP continued to increase (375.5 to 622.5 ng/mL) and liver metastases continued to enlarge, the third cycle of chemotherapy was replaced by VIP (third-line palliative chemotherapy: ifosfamide 2 g, cisplatin 120 mg, and etoposide 30 mg for 5 consecutive days). However, after the VIP chemotherapy, serum AFP had increased to 994.7 ng/mL. The patient underwent hepatic arteriography and embolization on 19 January 2020. From February 26 to April 21, she received radiotherapy for right liver metastases and took oral anlotinib (12 mg/d po for consecutive 14 days). The lesions in the right liver were significantly smaller than before. Positron emission tomography/computed tomography (PET/CT) showed that low-density glucose metabolism in the right lobe, near the diaphragmatic parietal region, was significantly increased. Then 5 cycles of tislelizumab (fourth-line palliative chemotherapy: 200 mg iv) targeted therapy were performed. Serum AFP fluctuation was shown in **Figure 2**.** A** new lesion appeared by CT scan in left liver on August 2020. Consequently, she received 2 cycles of radiotherapy + tislelizumab (200 mg iv) + vinorelbine tartrate capsules (60 mg po once a week). Unfortunately, serum AFP rose persistently and PET/CT showed active right liver metastases.

Then, she underwent a right hepatectomy at Beijing Nanjiao Tumor Hospital on December 3. The postoperative pathology was recurrence and metastasis of YST, without vascular tumor thrombus and nerve invasion. Both BRCA and MMR gene tests were negative. After the fourth surgery, she received TIP (fifth-line palliative chemotherapy, day 1: paclitaxel 250 mg/m^2^, 24-hour continuous infusion; day 1-3: ifosfamide 1,500 mg/m^2^, over 60 minutes’ infusion, and cisplatin 25 mg/m^2^, over 30 minutes’ infusion) adjunct chemotherapy for over four cycles. In the last two cycles of chemotherapy, bevacizumab was added intravenously because of the poorly decreased serum AFP. The patient began regular follow-up with serum AFP hovering around 400.0 ng/mL.

However, her serum AFP increased to 3550.0 ng/mL with enlarged left liver lesions and metastatic right iliac lymph nodes on 13 August 2021. The patient underwent left hepatic lesions and right iliac lymph nodes resection at Beijing Nanjiao Tumor Hospital on 28 September 2021. The postoperative pathology was the same as before. Then, she visited our hospital for further treatment.

**Figure 3:**
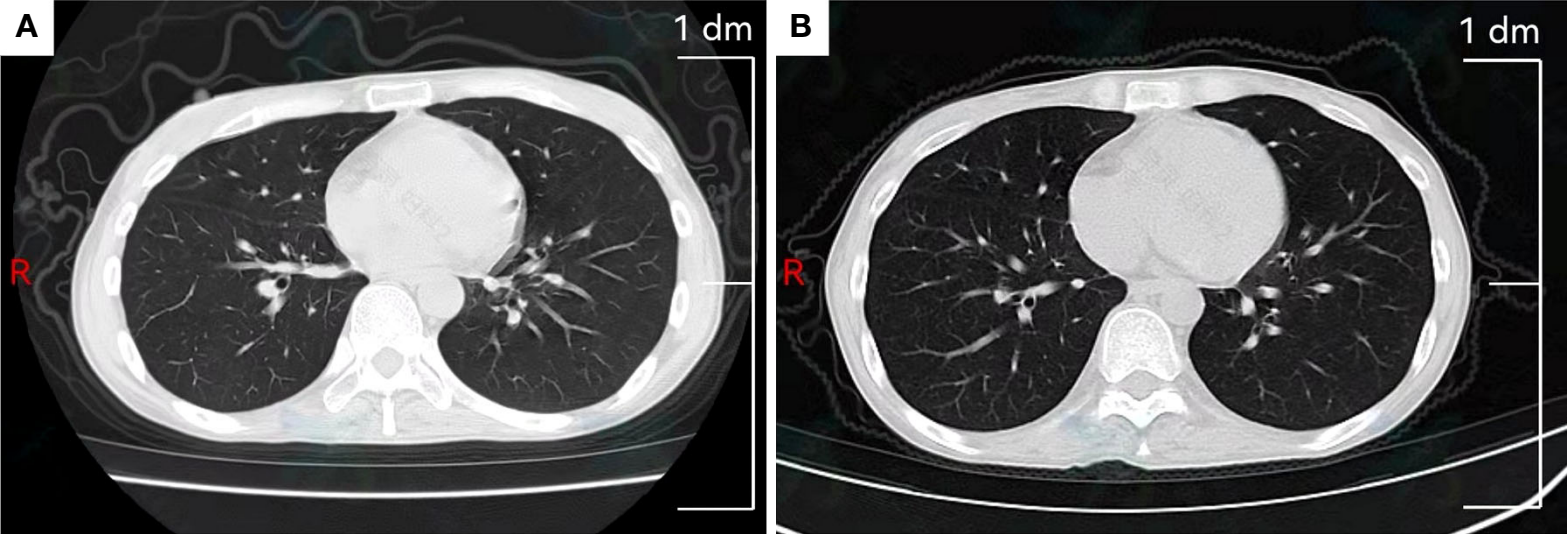
Chest computed tomography scan. **(A)** Slight interstitial changes in the lung before BEP´ chemotherapy. **(B)** Slight interstitial changes in the lung after BEP´ chemotherapy.

By reviewing previous diagnosis and treatments, conclusion was made that the patient had a better effect on the BEP chemotherapy. Therefore, we planned to implement 4 cycles of BEP´ (boanmycin, etoposide, and cisplatin) chemotherapy for the patient every 3 weeks. We replaced bleomycin with boanmycin, which had a lower rate of pulmonary fibrosis, and avoided the cumulative dose of bleomycin. The specific regimen was as follows: boanmycin (5~6 mg/m^2^ im for twice a week), etoposide (100 mg/m^2^ iv for 5 consecutive days), and cisplatin (15 mg/m^2^ iv for 5 consecutive days). After the first cycle, serum AFP was less than 30.0 ng/mL for the first time after relapse. Owing to multiple previous chemotherapies, she developed third-degree myelosuppression. In subsequent chemotherapy regimens, we reduced the doses of etoposide and cisplatin by 10%. However, due to a reduction in the chemotherapy dose and a prolonged interval between chemotherapies, serum AFP rose to 215.0 ng/mL. Comparing the chest CT scans before and after BEP´ chemotherapy, there was no significant change in the interstitium of the lung. (**Figure 3**). Currently, we plan to treat the patient with TP/TE (paclitaxel, cisplatin/paclitaxel, etoposide) chemotherapy every 3 weeks, but she is severely myelosuppression and poorly tolerated.

## Discussion

Primary extragonadal YST is a rare malignancy that seldom occurs in the peritoneum. Thirteen cases in primary peritoneal YST have been reported in literatures, including 10 cases in omentum (6, 7, 11, 12, 18–23) and 3 cases in mesentery (24, 25). To our knowledge, our case is the first primary relapsed and refractory peritoneal YST reported in the literature. We summarize the characteristics of these 13 cases in **Table 1**.

**Table 1:** Clinicopathologic features of reported cases of pelvic extragonadal YST.

Author [Reference]	Age (y), sex	Chief complaint	Primary Site	FIGO Stage	Tumor marker	Frozen biopsy	Treatment	Chemotherapy	Prognosis^a^	Follow-up (month)
Jones et al. (24)	2, male	Abdominal distension	Mesentery	IV	NR	ND	MTR, PJR, HDR	BEP	Poor	NED, 6
Jones et al. (24)	17, male	Abdominal pain	Mesentery	NA	AFP: 96^b^	ND	MTR, R&T-C	BEP	Good	NR
Park et al. (21)	45, female	Abdominal distension	Omentum	NA	AFP: 20,250^d^ CA125: WNL	YST	ICO, BSO, TAH	BEP	Poor	NED, 10
Xinghui et al. (11)	3, male	Abdominal distension	Omentum	NA	AFP: 1,210^d^ CA125: NR	YST (percutaneous needle biopsy)	Omentectomy	Neoadjuvant^c^	Intermediate	NR
Geminiani et al. (6)	46, female	Abdominal pain	Omentum	NA	AFP: 21,550^d^	PD malignant tumor	TO, TC, BSO, TAH, IS	BEP	Poor	NED, 24
Kim et al. (12)	37, female	Abdominal pain	Omentum	NA	AFP: 2,980^b^ CA125: 374	Adenocarcinoma or mesothelioma	SCO, BSO, TAH, P&PA-LND, MPB, appendectomy	BEP	Intermediate	NED, 12
Tangour-Bouaicha et al. (25)	16, female	Abdominal pain	Mesentery	IV	AFP: 21,000^b^	ND	MTR, HDR	BEP	Poor	NED, 21
Haibin et al. (19)	44, female	Abdominal distension	Omentum	NA	AFP: 27,612^d^	YST	ICO, BSO, TAH	BEP	Poor	NED, 7
Dulger et al. (22)	19, male	Abdominal dullness	Omentum	NA	AFP: 1,200^d^ CA125: 500	YST	Omentectomy	BEP	Intermediate	Died of cardiac arrest 15 days later
Harano et al. (18)	35, male	Abdominal distension	Omentum	NA	AFP: 7,144^e^	Colorectal tumor	Two courses of mFOLFOX6, omentectomy	Neoadjuvant: BEP	Intermediate	NED, 6
Ravishankar et al. (23)	34, female	Abdominal distension	Omentum	IIIC	AFP: 103,200^d^	YST	Omentectomy	BEP	Poor	NED, 10
Lim et al. (7)	32, female	Abdominal distension	Omentum	NA	AFP: 11,576^b^ CA125: 364	Poorly differentiated tumor	TO, BSO, TAH, P&PA-LND, appendectomy	BEP	Poor	NED, 48
Sudour-Bonnange et al. (20)	1, female	Abdominal pain	Omentum	NA	AFP: 52,000^d^	YST and IT	TO	VIP plus BCA	Poor	NED, 96
Present case	34, female	Abdominal pain	Mesentery	IV	AFP:228,499^d^ CA125: 167	ND	MTR, PS&S-LF; SR; HA&E; RH; LHLR, RI-LNR	BEP PC	Poor	NA

NR, not reported; ND, not done; MTR, mesenteric tumor resection; PJR, partial jejunum resection; HDR, Hepatic nodule resection; NED, no evidence of disease; NA, not available; R&T-C, right and transverse colectomy; ICO, infracolic omentectomy; BSO, bilateral salpingo-oophorectomy; TAH, total abdominal hysterectomy; PD, poorly differentiated; TO, total omentectomy; TC, total colectomy; IS, ileostomy; SCO, supracolic omentectomy; P&PA-LND, pelvic and paraaortic-lymph node dissection; MPB, multiple peritoneal biopsy; IT, immature teratoma; BCA, bleomycin, carboplatin and adriamicyn; PS, partial sigmoidectomy and single-lumen fistula of sigmoid colon; SR, stoma reversion of sigmoid colon; HA&E, hepatic arteriography and embolization; RH, right hepatectomy; LHLR, left hepatic lesion resection; RI-LNR, right iliac lymph node resection; PC, palliative chemotherapy.

a Definition of the germ cell consensus classification in reference (26).

b This level was checked postoperatively.

c Intravenous cyclophosphamide and arterial cisplatin + doxorubicin.

d This level was checked preoperatively.

e This level was checked after two courses of mFOLFOX6.

Like other extragonadal locations, the histogenesis of peritoneal YST is still controversial. Currently, three theories have been put forward to describe the origination of extra-ovarian germ cell tumors (6, 12, 21, 25). The most widely accepted hypothesis is that these tumors stem from the dislocation and stagnation of germ cells during embryonic development and migration. The primitive gonads migrate from the yolk sac to the genital ridge of the posterior peritoneum. It matures into ovaries or testes under the control of sex chromosome and then descends to the pelvis and scrotum, respectively. Meanwhile, the primordial gonads also undergo ectopic migration, mainly to the midline, such as the pineal gland, mediastinum, posterior peritoneum, and sacrococcygeal. The malignant transformation of these misplaced cells leads to primary extragonadal germ cell tumors. According to this hypothesis, the abnormal stagnation of germ cells in mesentery explains the origin of the tumor in our case. The second theory is abnormal differentiation of somatic cells. This is the case that YST occurs in the endometrium and stomach. The third mechanism is the metastasis of occult ovarian lesions. In our case, the patient’s ovaries were normal in imaging and intraoperative exploration, which ruled out this hypothesis.

The International Germ Cell Cancer Collaborative Group (IGCCCG) classification is widely used in the management of metastatic germ cell tumors. The system divides patients into good, intermediate, and poor prognosis groups based on the primary site, pretreatment tumor marker levels (AFP and beta-human chorionic gonadotropin), serum lactate dehydrogenase (LDH) levels, and disease severity. But data for this classification is obtained from patients treated between 1975 and 1990 (26). Recently, Gillessen S *et al.* publish the IGCCCG Update model which improves the prognosis of metastatic germ cell tumors. Furthermore, the model identifies a new cutoff of LDH with a 2.5× upper limit above normal and adds increasing age and lung metastasis as additional poor prognostic factors (27, 28). In our case, the postoperative pathology of the patient was mesenteric YST, invading the adventitia of the descending colon near the muscular layer, without lymph node metastasis. However, her preoperative serum AFP was 228,499.0 ng/mL, and we considered her prognosis to be poor.

Histopathologically, the appearance of extragonadal YST is similar to the ovarian ones with a marked architectural polymorphism. The reticular or microcystic pattern is the most common description, characterized by a loose, empty reticular structure in which tumor cells form intercommunicating cysts and tubular structures. Pseudopapillary/endodermal sinus, myxoma, hepatoid, glandular, and solid patterns are also observed (4–7, 21). Schiller-Duvall bodies are characteristics of YST but are only observed in 50% of ovarian sites (29). Cytopathologically, cohesive clusters or acinar structures composed of cells with vacuolar cytoplasm and macronuclei are observed in extragonadal YST. Extracellular matrix and clear globules can also be observed (29).

Immunohistochemistry is very helpful in confirming the diagnosis of YST. The most common immunohistochemical markers in YST are AFP, Glypican-3, SALL4, PLAP, and CKpan. SALL4 is a sensitive (100% sensitivity) marker for the diagnosis of extragonadal YST, more sensitive than PLAP, AFP, or glypican-3. At the same time, it is particularly useful in distinguishing YST from clear cell carcinoma (30, 31). Furthermore, ZBTB16 is highly sensitive and specific for YST and is superior to AFP and glypican-3 in the diagnosis of metastatic and extragonadal settings (32). Meanwhile, the diagnosis of germ cell tumors also requires specific serum tumor markers. Serum AFP has important value in the diagnosis, prognosis and treatment of YST. AFP is a glycoprotein normally produced by the yolk sac of the fetus, derived from the yolk sac or embryonic carcinoma component of germ cell carcinomas. Elevated serum AFP is commonly seen in hepatocellular carcinoma, liver inflammation due to cirrhosis or hepatitis, and embryonal tumors (33). Serum AFP levels are elevated in > 90% of YST (34), which in our case, it increased to 228,499.0 ng/mL. Serum AFP is an indicator for evaluating the chemotherapy efficacy and prognosis of YST (35, 36). The National Comprehensive Cancer Network (2016) recommends that patients who complete the clinical course be regularly monitored for AFP for 2 years (34). Notably, there is limited data on the diagnosis and treatment of postmenopausal YST because AFP is not routinely tested among this population, making correct diagnoses challenging. For this reason, their prognoses are poor, even for patients with early-stage YST. Therefore, YST should be suspected in postmenopausal women with ovarian masses and elevated serum AFP levels (37).

According to few cases reported about extragonadal YSTs, surgery plus chemotherapy is the main strategy. But treatment regimens are controversial due to the lack of standard guidelines (29, 38). For patients with YST without fertility requirements, comprehensive staging surgery is recommended. Four courses of BEP chemotherapy after surgery is preferred (39). Remarkably, the pulmonary toxicity of boanmycin is significantly lower than that of bleomycin, accounting for only 0.3% (1/325) (17). Some studies showed that patients with testicular germ cell tumors achieved good curative effects with VIP chemotherapy after surgery (40). In addition, Junpei et al. performed VIP chemotherapy in a patient with pancreatic YST with liver recurrence and achieved a complete pathological response (41). Kondagunta et al. showed that four cycles of TIP chemotherapy as second-line therapy achieved durable complete response rates in most patients with recurrent testicular germ cell tumors (42). For patients with poor response to initial chemotherapy, TIP chemotherapy is effective in germ cell tumor patients with relapsed or cisplatin-refractory diseases (43). Patients with recurrent metastatic germ cell tumors were also cured by high-dose chemotherapy (HDCT) and peripheral blood stem cell transplantation (PBSCT) in third-line or subsequent therapies (44, 45). In conclusion, VIP chemotherapy, TIP chemotherapy, HDCT, and PBSCT, being second-line treatment and above, improved the prognosis of recurrent germ cell tumors. For patients with limited recurrent tumor sites, secondary cytoreductive surgery may benefit them (46).

We reported a case of refractory mesenteric YST in a 37-year-old woman benefiting from BEP´ chemotherapy regimen. In this case, boanmycin substituted bleomycin, which solved problem of the cumulative dose of bleomycin and reduced the occurrence of pulmonary toxic side effects.

## Data availability statement

The original contributions presented in the study are included in the article/supplementary material. Further inquiries can be directed to the corresponding author.

## Ethics statement

This study was performed in accordance with the Declaration of Helsinki and approved by the Ethics Committee of the First Affiliated Hospital of Xi’an Jiaotong University (XJTU1AF2022LSK-194).

## Author contributions

QL design the study and perform the chemotherapy. XZ, LZ, XF, ZP, ML, MG, and HH collected the data. XZ, LZ, YB, and SZ prepared the manuscript. All authors contributed to the article and approved the submitted version.

## Conflict of interest

The authors declare that the research was conducted in the absence of any commercial or financial relationships that could be construed as a potential conflict of interest.

## Publisher’s note

All claims expressed in this article are solely those of the authors and do not necessarily represent those of their affiliated organizations, or those of the publisher, the editors and the reviewers. Any product that may be evaluated in this article, or claim that may be made by its manufacturer, is not guaranteed or endorsed by the publisher.
